# Randomized controlled phase III trial to investigate superiority of robot-assisted gastrectomy over laparoscopic gastrectomy for clinical stage T1-4aN0-3 gastric cancer patients (JCOG1907, MONA LISA study): a study protocol

**DOI:** 10.1186/s12885-023-11481-2

**Published:** 2023-10-17

**Authors:** Rie Makuuchi, Masanori Terashima, Mitsumi Terada, Junki Mizusawa, Ryosuke Kita, Masanori Tokunaga, Takeshi Omori, Toshiyasu Ojima, Kazuhisa Ehara, Masaya Watanabe, Yoshitomo Yanagimoto, Souya Nunobe, Takahiro Kinoshita, Seiji Ito, Yasunori Nishida, Jun Hihara, Narikazu Boku, Yukinori Kurokawa, Takaki Yoshikawa, the Stomach Cancer Study Group of Japan Clinical Oncology Group

**Affiliations:** 1 Department of Gastroenterological Surgery, Cancer Institute Hospital of Japanese Foundation for Cancer Research, 3-8-31, Ariake, Koto-ku, Tokyo, 135-8550 Japan; 2 Division of Gastric Surgery, Shizuoka Cancer Center, 1007, Shimonagakubo, Nagaizumi-Cho, Sunto-Gun, Shizuoka, Japan; 3 Department of International Clinical Development, National Cancer Center Hospital, Tokyo, Japan; 4 Japan Clinical Oncology Group Data Center/Operations Office, National Cancer Center Hospital, Tokyo, Japan; 5 Department of Gastrointestinal Surgery, Tokyo Medical and Dental University, Tokyo, Japan; 6 Department of Gastroenterological Surgery, Osaka International Cancer Institute, Osaka, Japan; 7 Second Department of Surgery, Wakayama Medical University Scholl of Medicine, Wakayama, Japan; 8 Department of Gastroenterological Surgery, Saitama Cancer Center, Saitama, Japan; 9 Department of Gastroenterological Surgery, Shizuoka General Hospital, Shizuoka, Japan; 10 Department of Surgery, Toyonaka Municipal Hospital, Osaka, Japan; 11 Department of Gastric Surgery, National Cancer Center Hospital East, Kashiwa, Japan; 12 Department of Gastroenterological Surgery, Aichi Cancer Center Hospital, Nagoya, Japan; 13 Department of Surgery, Keiyukai Sapporo Hospital, Hokkaido, Japan; 14 Department of Surgery, Hiroshima City North Medical Center Asa Citizens Hospital, Hiroshima, Japan; 15 Department of Oncology and General Medicine, IMSUT Hospital, The Institute of Medical Science, The University of Tokyo, Tokyo, Japan; 16 Department of Gastroenterological Surgery, Osaka University Graduate School of Medicine, Osaka, Japan; 17 Department of Gastric Surgery, National Cancer Center Hospital, Tokyo, Japan

**Keywords:** Stomach neoplasms, Robotic surgery, Minimally invasive surgery, Postoperative complication, Multicenter study, Randomized controlled trial

## Abstract

**Background:**

Laparoscopic gastrectomy (LG) is considered a standard treatment for clinical stage I gastric cancer. Nevertheless, LG has some drawbacks, such as motion restriction and difficulties in spatial perception. Robot-assisted gastrectomy (RG) overcomes these drawbacks by using articulated forceps, tremor-filtering capability, and high-resolution three-dimensional imaging, and it is expected to enable more precise and safer procedures than LG for gastric cancer. However, robust evidence based on a large-scale randomized study is lacking.

**Methods:**

We are performing a randomized controlled phase III study to investigate the superiority of RG over LG for clinical T1-2N0-2 gastric cancer in terms of safety. In total, 1,040 patients are planned to be enrolled from 46 Japanese institutions over 5 years. The primary endpoint is the incidence of postoperative intra-abdominal infectious complications, including anastomotic leakage, pancreatic fistula, and intra-abdominal abscess of Clavien–Dindo (CD) grade ≥ II. The secondary endpoints are the incidence of all CD grade ≥ II and ≥ IIIA postoperative complications, the incidence of CD grade ≥ IIIA postoperative intra-abdominal infectious complications, relapse-free survival, overall survival, the proportion of RG completion, the proportion of LG completion, the proportion of conversion to open surgery, the proportion of operation-related death, and short-term surgical outcomes. The Japan Clinical Oncology Group Protocol Review Committee approved this study protocol in January 2020. Approval from the institutional review board was obtained before starting patient enrollment in each institution. Patient enrollment began in March 2020. We revised the protocol to expand the eligibility criteria to T1-4aN0-3 in July 2022 based on the results of randomized trials of LG demonstrating non-inferiority of LG to open surgery for survival outcomes in advanced gastric cancer.

**Discussion:**

This is the first multicenter randomized controlled trial to confirm the superiority of RG over LG in terms of safety. This study will demonstrate whether RG is superior for gastric cancer.

**Trial registration:**

The protocol of JCOG1907 was registered in the UMIN Clinical Trials Registry as UMIN000039825 (http://www.umin.ac.jp/ctr/index.htm). Date of Registration: March 16, 2020. Date of First Participant Enrollment: April 1, 2020.

## Background

The Stomach Cancer Study Group (SCSG) of the Japan Clinical Oncology Group (JCOG) previously established evidence of the safety and efficacy of laparoscopic gastrectomy (LG) for clinical stage I (cT1N0/T1N1/T2N0) gastric cancer. JCOG0912, a randomized controlled trial, confirmed the non-inferiority of laparoscopic distal gastrectomy (LDG) to open distal gastrectomy (ODG) in terms of relapse-free survival (RFS) [1]. JCOG1401, a single-arm confirmatory trial, also showed the safety of laparoscopic total gastrectomy (LTG) and proximal gastrectomy (LPG) for patients with clinical stage I gastric cancer [2]. Based on these results, LG is recommended as a standard treatment for clinical stage I gastric cancer in the Japanese Gastric Cancer Treatment Guidelines [3].

To confirm the safety and efficacy of LDG for advanced gastric cancer, randomized controlled trials have been conducted in Japan (JLSSG0901), Korea (KLASS-02), and China (CLASS-01). With respect to short-term outcomes, the incidence of postoperative complications was not different between LDG and ODG in CLASS-01 [4], whereas it was significantly lower in LDG than ODG in KLASS-02 [5]. In terms of survival, only the result of CLASS-01 was available at the time the present study was designed, and it showed the non-inferiority of LDG to ODG regarding 3-year RFS [6].

Because surgical procedures and results such as the operation time and blood loss may differ slightly between Japan and other countries, the efficacy of LG for advanced gastric cancer was debated in Japan until the survival outcomes of Japanese trials were obtained. The SCSG of the JCOG reached a consensus that LG should be a standard treatment for clinical T1-2N0-2 gastric cancer because the target of JCOG0912 and JCOG1401 was clinical T1N0/T1N1/T2N0 in the 13th edition of the Japanese Classification of Gastric Carcinoma, which is not significantly different from surgery for clinical T1-2N0-2 in the 15th edition (current classification).

Although the indications for LG are expanding, LG has some drawbacks that must be overcome, such as motion restriction using straight forceps and difficulties in spatial perception due to the use of two-dimensional imaging. Real-world data indicate that compared with open gastrectomy, LDG is associated with a higher incidence of pancreatic fistula [7] and that LTG is associated with a higher incidence of anastomotic leakage [8].

Surgical robots are equipped with articulated forceps, tremor-filtering capability, and high-resolution three-dimensional images. Therefore, robot-assisted gastrectomy (RG) is expected to overcome the drawbacks of LG and enable performance of more meticulous surgery. Many retrospective studies have been conducted, and a meta-analysis of these studies revealed that RG was associated with fewer postoperative complications, especially pancreas-related complications; an increased number of harvested lymph nodes; an earlier time to first flatus; and less intraoperative blood loss [9]. Additionally, a multi-institutional prospective study showed the safety of RG for clinical stage I and II gastric cancer [10]. However, that was a single-arm study comparing LG as the historical control. Validation by randomized controlled trials is therefore necessary.

The SCSG of the JCOG designed a multicenter randomized controlled phase III trial to investigate the superiority of RG over LG for clinical T1-2N0-2 gastric cancer (JCOG1907, MONA LISA study). During enrollment of patients in this study, the results of KLASS-02 [11] and JLSSG0901 [12] were published, demonstrating non-inferiority of LDG to ODG with respect to survival outcomes in patients with advanced gastric cancer. Based on these results, we revised the protocol for eligible patients from clinical T1-2N0-2 to T1-4aN0-3 in July 2022.

## Methods/design

### Aim

This study aims to confirm the superiority of RG over LG in terms of safety for patients with clinical T1-4aN0-3 gastric cancer.

### Study setting

This is a multi-institutional, two-arm, open-label, randomized phase III trial.

### Endpoints

The primary endpoint is the incidence of postoperative intra-abdominal infectious complications (IAIC), defined as Clavien–Dindo (CD) [13, 14] grade ≥ II complications. IAIC includes anastomotic leakage, pancreatic fistula, and intra-abdominal abscess. The secondary endpoints are the incidences of all postoperative complications of CD grade ≥ II and ≥ IIIA, the incidence of IAIC of CD grade ≥ IIIA, RFS, overall survival (OS), the proportion of RG completion, the proportion of LG completion, the proportion of conversion to open surgery, the proportion of operation-related death, and short-term surgical outcomes.

All postoperative complications, including IAIC, are defined as those that develop within 30 days after surgery among all patients undergoing operations. OS is defined as the time from randomization to death of any cause and will be censored on the last day of contact for a surviving patient. RFS is defined as the time from randomization to relapse or death of any cause and will be censored on the last day of contact when the patient is alive with no evidence of relapse. The proportion of RG completion is defined as the proportion of patients for whom RG is completed without conversion to open or laparoscopic surgery among all patients undergoing operations in the RG arm. The proportion of LG completion is defined as the proportion of patients for whom LG is completed without conversion to open surgery among all patients undergoing operations in the LG arm. The proportion of conversion to open surgery is defined as the proportion of patients who undergo open surgery among all patients undergoing operations in each group. The short-term surgical outcomes are (i) the time from the end of surgery to the first episode of flatus and (ii) the proportion of patients requesting an analgesic on postoperative days 5 to 10.

### Eligibility criteria

#### Inclusion criteria

1. The cancer is histologically proven adenocarcinoma of the stomach.
2. The cancer is clinical T1-T4a and N0-3 according to the Japanese Classification of Gastric Carcinoma (15th edition).
3. The cancer has been diagnosed as H0P0M0 with no bulky metastatic lymph nodes.
4. In cases of T2 or deeper cancer in the upper third of the stomach, the tumor does not involve the greater curvature.
5. There is no indication for endoscopic mucosal resection (EMR) or endoscopic submucosal dissection (ESD).
6. For patients who have previously undergone EMR or ESD, all of the following conditions must be fulfilled:

- Pathological findings indicate the requirement for additional gastrectomy.
- EMR or ESD was performed ≤ 91 days previously.
- Perforation by EMR or ESD is not considered.

7) R0 resection is expected.
8) Neither a Borrmann type 4 tumor nor a large (≥ 8-cm) type 3 tumor is present.
9) No invasion to the esophagus or duodenum is present.
10) The cancer is not remnant gastric cancer.
11) The patient is ≥ 20 years of age.
12) The patient has an Eastern Cooperative Oncology Group performance status of 0 or 1.
13) The patient has a body mass index of < 30 kg/m^2^.
14) The patient has no history of upper abdominal surgery except for laparoscopic cholecystectomy.
15) No prior abdominal radiation therapy was received for any other malignancies. Patients with a history of chemotherapy and/or hormone therapy are eligible.
16) Sufficient organ function is confirmed within 28 days of registration:

- White blood cell count of ≥ 3000/mm^3^.
- Platelet count of ≥ 10 × 10^4^/mm^3^.
- Total bilirubin concentration of ≤ 2.0 mg/dL.
- Aspartate aminotransferase concentration of ≤ 100 IU/L.
- Alanine aminotransferase concentration of ≤ 100 IU/L.
- Creatinine concentration of ≤ 1.5 mg/dL.

17) Written informed consent is provided.

#### Exclusion criteria

1. Synchronous or metachronous (within 5 years) malignancies except cancer with a 5-year relative survival rate of ≥ 95%, such as carcinoma in situ, intramucosal tumor, or early-stage cancers.
2. Infectious disease requiring systemic treatment.
3. Body temperature of ≥ 38 °C.
4. Women who are pregnant, possibly pregnant, within 28 days after delivery, or breastfeeding; men who wish for their partner to become pregnant.
5. Severe psychiatric disease.
6. Continuous systemic steroids or immunosuppressive drug therapy.
7. Unstable angina pectoris (angina developed or attack worsened within the previous 3 weeks) or myocardial infarction within the previous 6 months.
8. Poorly controlled valvular heart disease, dilated cardiomyopathy, or hypertrophic cardiomyopathy.
9. Positive for HIV antibody.
10. Severe interstitial pneumonia, severe lung fibrosis, or severe emphysema.

### Randomization

After confirming the eligibility criteria, registration to the JCOG Data Center will be performed using a web-based system. Patients will be randomized to either the LG arm or the RG arm by the minimization method with a random component balancing the arm with the institution, clinical stage (cT1N0/ cT1N1-3 or cT2-4aN0-3), and type of gastrectomy [DG or pylorus-preserving gastrectomy (PPG) / TG or PG].

### Treatments

LG or RG will be performed in each respective arm. All procedures will be identical except for the surgical approach. The extent of lymph node dissection will be decided according to the clinical T and N stages based on the 5th version of the Gastric Cancer Treatment Guidelines in Japan. D1 + dissection will be applied for cT1N0 tumors, and D2 dissection will be applied for cT2-4a or cN1-3 tumors. PPG and PG will be allowed only for cT1N0 tumors. Splenectomy or bursectomy will not be allowed. Omentectomy will be required for cT3-T4a tumors. Mini-laparotomy should be limited to one site, and the length of the skin incision should be ≤ 6 cm. When the skin incision requires extension by > 6 cm, conversion to open surgery will be considered. Intraoperative lavage cytology will be required for cT2-4a tumors. If the intraoperative findings reveal a T4b or stage IVB tumor, the protocol treatment will be terminated; subsequent treatment for the patient is not specified.

### Surgical robot

The da Vinci Surgical System by Intuitive Surgical (Sunnyvale, CA, USA) will be used for the RG arm because it was the only surgical robot available in Japan at the time of protocol development. However, if other surgical robots become available for clinical use during the study, their availability will be discussed and reviewed in the SCSG of the JCOG.

### Quality control of study

The principal investigator will designate a certified surgeon in charge of the study according to the criteria outlined below. The SCSG chair of the JCOG will designate a study-specific surgical quality assurance (QA) committee.

#### Certified surgeon for LG

In the LG arm, surgeons must fulfill the following criteria according to the degree of lymphadenectomy or type of gastrectomy: for any type of gastrectomy with D1 + lymphadenectomy, the surgeon must fulfill (1) and (2); for DG with D2 lymphadenectomy, the surgeon must fulfill (1) to (3); and for TG with D2, the surgeon must fulfill (1) to (4).

1. Experience in performing ≥ 30 LG procedures.
2. Certification in the field of gastric cancer by either a study-specific surgical QA committee or the Japan Society for Endoscopic Surgery (JSES).
3. Experience in performing ≥ 20 LG procedures with D2 lymphadenectomy.
4. Experience in performing ≥ 15 laparoscopic esophagojejunal anastomoses.

#### Certified surgeon for RG

In addition to the criteria in the LG arm, all the following criteria must be fulfilled for certified surgeons in the RG arm.

1. Experience in performing ≥ 10 RG procedures, at least one of which must be TG or PG.
2. Certification as an RG proctor by the JSES or the study-specific surgical QA committee.

A certified surgeon will perform LG and RG as an operator. The certified surgeon can be a teaching assistant for RG with D1 + lymphadenectomy using a dual console or for LG with D1 + lymphadenectomy.

#### Intraoperative photographs and video recording

The study coordinator will conduct a central peer review of the surgical procedure every 6 months by checking the intraoperative photograph of the surgical field from all registered patients. Intraoperative videos of arbitrary patients will also be shown in a group conference held three times yearly to share the surgical procedure.

### Follow-up

After curative resection, adjuvant chemotherapy with S1 for 1 year will be recommended for patients with pathological stage II cancer, and S1 plus docetaxel for 1 year will be recommended for those with pathological stage III cancer. The post-study treatment is not specified for patients with non-curative resection or pathological stage IV cancer.

All randomized patients will be followed up for at least 5 years. Tumor marker measurement, chest X-ray examination, and enhanced computed tomography examination will be performed at least every year, and upper gastrointestinal endoscopy will be performed every 2 years for the duration of follow-up for patients with pathological stage I cancer. For patients with pathological stage II and III cancer, the follow-up schedule is as follows: tumor markers will be evaluated every 3 months for the first 2 years and every 6 months for the next 3 years; enhanced computed tomography will be performed every 6 months for the first 3 years, and for the next 2 years, chest X-ray examination will be performed every year; and upper gastrointestinal endoscopy will be performed every 2 years.

### Study design and statistical analysis

This randomized study is designed to demonstrate that RG is superior to LG in terms of safety. RFS and OS are secondary endpoints to confirm that RG is not inferior to LG in terms of long-term survival. When the data demonstrate superiority of the safety of RG over LG, RG will be considered one of the treatment options for gastric cancer. In addition, if RFS is confirmed to be non-inferior in RG versus LG, the SCSG of the JCOG will consider RG the new standard treatment.

In JCOG0912 and JCOG1401, the incidence of IAIC (CD grade ≥ II) was 2.6% for LDG/LPPG with D1+, 6.7% for LDG with D2, 7.8% for LTG/LPG with D1+, and 20.0% for LTG with D2. We assume that the distribution of the type of gastrectomy and degree of lymphadenectomy in the present study will be as follows: DG/PPG with D1+: 50%, DG with D2: 17%, TG/PG with D1+: 25%, and TG with D2: 8%. Based on the above, the incidence of IAIC in the LG arm is expected to be approximately 6.0%. We anticipate that the use of the robot will halve the incidence of IAIC to 3% according to the results of previous prospective studies [10].

With a one-sided alpha of 5%, 1,006 patients will be required to preserve the power of 70%. The total sample size is set at 1,040 patients, assuming that a few patients would not undergo gastrectomy. All statistical analyses will be conducted at the JCOG Data Center.

### Interim analysis and monitoring

We plan to conduct one interim analysis, considering multiplicity using the Lan–DeMets method with the O’Brien–Fleming type alpha spending function [15]. The Data and Safety Monitoring Committee of the JCOG will independently review the interim analysis reports and recommend early termination of the trial if necessary. In-house monitoring will be performed every 6 months by the JCOG Data Center to evaluate and improve the study progress, data integrity, and patient safety.

## Discussion

This study aims to confirm the superiority of RG over LG for gastric cancer. Unless a clear advantage of robot-assisted surgery is demonstrated, it cannot be recommended as standard treatment because the cost of RG is much higher than that of LG [9].

RG is expected to overcome the drawbacks of LG, such as movement restrictions and difficulties in spatial perception, which would trigger IAIC. Thus, we are adopting the incidence of CD grade ≥ II IAIC as the primary endpoint. IAIC is a significant burden for the patient because it requires treatment with antimicrobials, drainage, and extended hospitalization. In addition, IAIC can lead to pseudoaneurysm formation, which can cause intra-abdominal bleeding and death. Moreover, recent studies have shown a negative impact of postoperative complications on long-term survival outcomes. We previously reported that CD grade ≥ II IAIC adversely affected on OS [16] using data from JCOG1001, which compared bursectomy and non-bursectomy for patients with cT3/4a locally advanced gastric cancer. A meta-analysis showed that IAIC was a poor prognostic factor in gastric and other cancers [17].

If the issues of surgical time and medical costs have been resolved or alleviated at the end of this study, the results will be carefully interpreted and discussed within the SGCS of the JCOG. Ancillary analyses of surgeon fatigue and postoperative patient-reported outcomes, including cost-effectiveness, are also planned.

The real benefit of RG can be evaluated from this study because it is the only multicenter randomized controlled trial to confirm the superiority of RG.

## Data Availability

Data sharing is not applicable to this article because no datasets had been generated or analyzed at the time of submission.

## Abbreviations

SCSG: Stomach Cancer Study Group
JCOG: Japan Clinical Oncology Group
LG: laparoscopic gastrectomy
LDG: laparoscopic distal gastrectomy
ODG: open distal gastrectomy
RFS: relapse-free survival
LTG: laparoscopic total gastrectomy
LPG: laparoscopic proximal gastrectomy
RG: robot-assisted gastrectomy
IAIC: intra-abdominal infectious complications
CD: Clavien–Dindo classification
EMR: endoscopic mucosal resection
ESD: endoscopic submucosal dissection
PPG: pylorus-preserving gastrectomy
QA: quality assurance
JSES: Japan Society for Endoscopic Surgery
